# Painful fissured plaques on the palms and soles in a patient with chronic myeloid leukemia

**DOI:** 10.1016/j.jdcr.2026.01.020

**Published:** 2026-01-27

**Authors:** Pooja A. Shet, Anne Sompayrac, Janmesh D. Patel, Sara E. Dahle, Roslyn Rivkah Isseroff

**Affiliations:** a Dermatology Section, VA Northern California Health Care System, Sacramento VA Medical Center, Mather, California; b Department of Dermatology, UC Davis Health, Sacramento, California; c Podiatry Section, Surgical Services, VA Northern California Health Care System, Sacramento VA Medical Center, Mather, California

**Keywords:** acute drug reaction, asciminib, CML, hand-foot skin reaction, HFSR, imatinib, palmar-plantar erythrodysesthesia, skin of color, TKI inhibitor

## Case presentation

**Fig 1:**
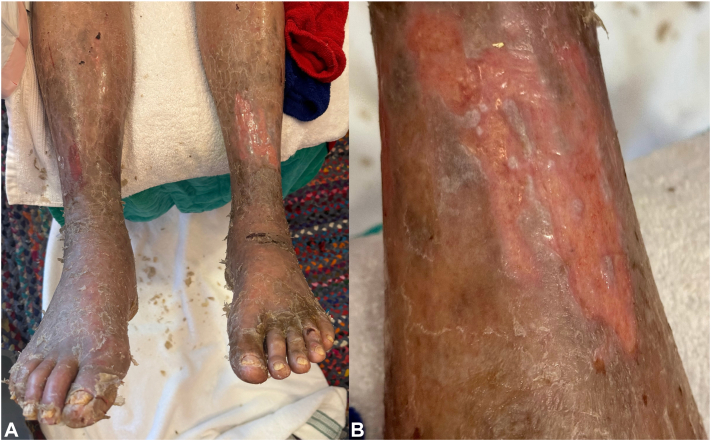
**A,** Dorsal view of the feet approximately 2 weeks after starting asciminib, demonstrating diffuse hyperkeratotic plaques on the feet and shins with secondary fissuring and desquamation. **B,** Close-up view of the left shin 2 weeks after starting asciminib, showing hyperkeratosis with overlying scale and marked desquamation. Extension of scale and skin exfoliation to the shins is not classically seen in HFSR, demonstrating a nonclassical clinical presentation of the disease. *HFSR*, Hand-foot skin reaction.

**Fig 2:**
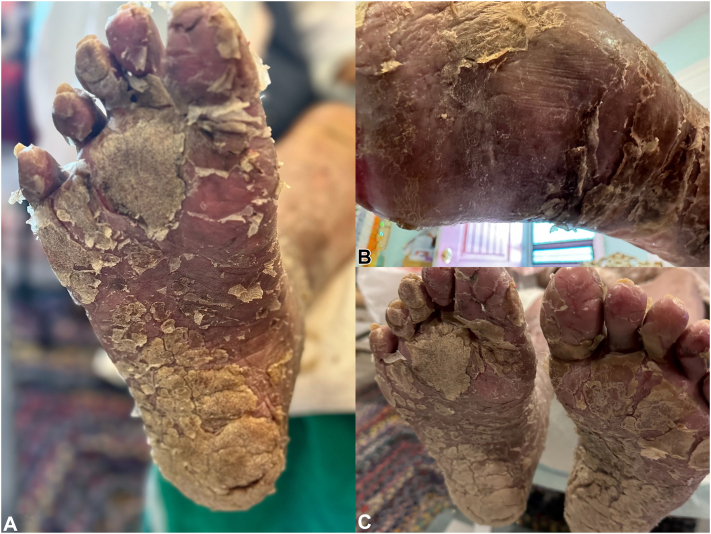
**A-C** Clinical photograph obtained of the plantar view of the feet approximately 2 weeks after starting asciminib. Images demonstrate hyperkeratotic plaques on an underlying erythematous base, with overlying xerosis, fissuring, and thick scale.

A 78-year-old male with chronic myeloid leukemia (CML) presented for intermittent eruption on the palms and soles for the past 14 months. Eighteen months prior, the patient began 100 mg daily imatinib therapy for CML, with subsequent dose increase to 200 mg daily, and then 400 mg daily, 3 and 6 months later, respectively. He reported onset of exquisitely painful fissured plaques on the bilateral hands and feet approximately 2 weeks after the imatinib dose increase, which subsequently required hospitalization and extensive home health wound care on discharge. His symptoms improved to near-complete resolution after discontinuation of imatinib. He began taking asciminib 40 mg daily 4 months after discontinuation of imatinib. Approximately 2 weeks after starting asciminib, painful fissured plaques reappeared on his feet and hands (Figs 1 and 2). There was no mucosal involvement. Topical triamcinolone acetonide 0.1% ointment daily, topical clobetasol propionate 0.05% ointment twice daily, and daily topical clotrimazole 1% cream were ineffective. After stopping asciminib, the eruption on the patient’s hands and feet began to gradually improve.

On presentation in clinic 2 weeks after discontinuation of the asciminib, a total body skin examination revealed macules and patches of postinflammatory hyperpigmentation and faint scale on the patient’s bilateral palms and soles. The bilateral lower extremities demonstrated pitting edema to the mid-shin. The right lower extremity demonstrated linear fissuring on the distal dorsal surface. Ankle-brachial index performed in clinic showed L: 1.09, R: 1.02. Potassium hydroxide test performed in clinic was negative.

**Question 1: What is the most likely diagnosis?**

**A.** Palmoplantar psoriasis
**B.** Tinea pedis with id reaction
**C.** Palmoplantar keratoderma
**D.** Hand-foot skin reaction
**E.** Stevens-Johnson syndrome

## Discussion

D. Hand-foot skin reaction–Correct. Hand-foot skin reaction (HFSR) is associated with tyrosine kinase inhibitor treatment, presenting as painful, well-demarcated erythematous plaques and blisters on areas of pressure or friction, often progressing to fissuring and callus formation.^1^ The temporal association with imatinib and asciminib, severe painful fissures of the palms and soles, hyperkeratosis, and improvement with drug discontinuation support a diagnosis of HFSR. While HFSR was originally thought to be caused solely by multikinase inhibitors, such as sorafenib and sunitinib, it has been seen with more selective tyrosine kinase inhibitors, such as imatinib and asciminib.^2^^,^^3^ In this patient case, the eruption was first caused by imatinib and subsequently reinduced by asciminib. Asciminib is a fourth-generation ABL kinase inhibitor, also known as a STAMP (Specifically Targeting the ABL Myristoyl Pocket) inhibitor, within the broader family of breakpoint cluster region protein-ABL inhibitors used to treat CML.^4^^,^^5^ Unlike earlier generations which block the ATP-binding site, asciminib binds to an allosteric myristoyl pocket distinct from the ATP-binding site, conferring distinct pharmacologic properties. It was approved by the Food and Drug Administration in October 2021 following the phase III ASCEMBL trial, which demonstrated superior efficacy compared to bosutinib in patients with CML.^5^ Notably, this patient’s eruption extended to the shins, a distribution not typical of classic HFSR.^1^

## Conflicts of interest

None disclosed.
